# A Real Time Chemotaxis Assay Unveils Unique Migratory Profiles amongst Different Primary Murine Macrophages

**DOI:** 10.1371/journal.pone.0058744

**Published:** 2013-03-14

**Authors:** Asif J. Iqbal, Daniel Regan-Komito, Ivy Christou, Gemma E. White, Eileen McNeill, Amy Kenyon, Lewis Taylor, Theodore S. Kapellos, Edward A. Fisher, Keith M. Channon, David R. Greaves

**Affiliations:** 1 Sir William Dunn School of Pathology, University of Oxford, Oxford, United Kingdom; 2 Department of Cardiovascular Medicine, University of Oxford, Oxford, United Kingdom; 3 NYU School of Medicine, Division of Cardiology, Department of Medicine, and the Marc and Ruti Bell Program in Vascular Biology, New York, New York, United States of America; McMaster University, Canada

## Abstract

Chemotaxis assays are an invaluable tool for studying the biological activity of inflammatory mediators such as CC chemokines, which have been implicated in a wide range of chronic inflammatory diseases. Conventional chemotaxis systems such as the modified Boyden chamber are limited in terms of the data captured given that the assays are analysed at a single time-point. We report the optimisation and validation of a label-free, real-time cell migration assay based on electrical cell impedance to measure chemotaxis of different primary murine macrophage populations in response to a range of CC chemokines and other chemoattractant signalling molecules. We clearly demonstrate key differences in the migratory behavior of different murine macrophage populations and show that this dynamic system measures true macrophage chemotaxis rather than chemokinesis or fugetaxis. We highlight an absolute requirement for Gαi signaling and actin cytoskeletal rearrangement as demonstrated by Pertussis toxin and cytochalasin D inhibition. We also studied the chemotaxis of CD14^+^ human monocytes and demonstrate distinct chemotactic profiles amongst different monocyte donors to CCL2. This real-time chemotaxis assay will allow a detailed analysis of factors that regulate macrophage responses to chemoattractant cytokines and inflammatory mediators.

## Introduction

The process of monocyte and macrophage recruitment to sites of tissue injury, inflammation or infection is essential for mounting effective immune responses and restoring immune homeostasis. Chemokines, a family of secreted signalling proteins made at sites of tissue damage, act as powerful chemoattractants for different sets of host immune cells by interacting with a series of specific G protein coupled receptors expressed on the cell surface [1], [2]. Macrophages play an active role in the initiation and resolution of acute inflammation [3], but can also orchestrate harmful processes associated with chronic inflammation. Failure to turn off the powerful CC chemokine signalling system in diseases such as rheumatoid arthritis results in the accumulation of activated macrophages and development of chronic inflammation [4]–[7]. For this reason many groups have been developing methods to block CC chemokine activity to reduce monocyte and macrophage accumulation at sites of chronic inflammation [8]–[12].

*In vitro* chemotaxis assays such as the modified Boyden chamber, Dunn chamber and under agarose have been used to measure the chemotactic capacity of various cell types toward a range of chemoattractants and for developing novel reagents to inhibit cell migration [13]–[15]. However, there are a number of limitations associated with these traditional assays which include; single time-point (Boyden chamber), low throughput, time-consuming quantification methods and the use of chemical or fluorescent labels. Recently ACEA in partnership with Roche Applied Science have developed the xCELLigence system for real-time label free monitoring of cellular migration through the incorporation of cell electrical impedance, a technology first described in the early 1990's [16].

In this study we validated the Real-Time Cell Analyser-Dual Plate (RTCA-DP) instrument and optimised assays for monitoring real-time chemotaxis of several primary murine macrophage populations. We highlight key differences amongst resident, thioglycollate elicited, Bio-Gel elicited and bone marrow derived macrophage populations in terms of their chemotaxis in response to a wide range of inflammatory mediators.

## Materials and Methods

### Materials

All cell culture media and buffers were obtained from PAA systems (Yeovil, UK) unless otherwise specified. All laboratory chemicals were obtained from Sigma-Aldrich (Gillingham, Dorset, UK). Chemokines and other chemoattractants were purchased from Peprotech (London, UK) and R&D Systems (Abingdon, Oxford, UK). Pharmacological inhibitors were purchased from Tocris (Bristol, UK) and Sigma-Aldrich.

### Bio-Gel and thioglycollate elicitation of primary mouse macrophages

All animal studies were conducted with ethical approval from the Dunn School of Pathology Local Ethical Review Committee and in accordance with the UK Home Office regulations (Guidance on the Operation of Animals, Scientific Procedures Act, 1986). Male C57BL/6J mice (Harlan Laboratories, Oxfordshire, UK) or ChemR23^−/−^ mice on an Sv129Ev background (a kind gift of Takeda Cambridge, U.K.) were injected intraperitoneally with 1 ml of sterile 2% Bio-Gel P-100 fine polyacrylamide beads (45–90 µm; BIO-RAD Laboratories, Hemel Hempstead, Hertfordshire, UK) suspended in PBS. In some experiments 1 ml of 4% Thioglycollate (Brewer Thioglycollate media; Sigma-Aldrich) prepared as previously described [17], was injected intraperitoneally. Mice were sacrificed after 4 days and the peritoneum lavaged with 10 ml of ice cold PBS/2 mMEDTA.

### Murine bone marrow derived macrophages

Bone marrow derived macrophages (BMDM) were generated as previously described [18]. Briefly, fresh bone marrow cells from tibiae and femurs of C57BL/6J mice (8–12 weeks) were flushed and cultured in DMEM supplemented with 10% heat-inactivated fetal bovine serum (FBS), 2 mM l-glutamine, 100 U/ml penicillin and 15% supernatant from L929 cells as a source of macrophage colony stimulating factor [19]. BMDM were generated by culturing 4×10^6^ bone marrow cells in 10 ml of medium in 100 mm petri dishes (Sterilin, Abergoed UK). On day 3, an additional 5 ml of medium was added. Cells were harvested with PBS/5 mM EDTA on day 7.

### Human monocyte isolation

Blood from healthy anonymous donors was obtained from the National Blood Services with appropriate local ethical approval. For monocyte isolation, blood was diluted 1∶3 with PBS and separated by ficoll gradient centrifugation as previously described [20]. Following PBMC isolation and washing, a total of 1.25×10^8^ PBMCs were labelled and positively selected with CD14 micro-beads and MACS separation (Miltenyi Biotec, Bisley, Surrey, UK). Monocytes were resuspended in chemotaxis buffer (RPMI 1640/25 mM HEPES/0.5% (w/v) BSA) and left on ice until required. Flow cytometry with an anti-CD14 antibody (AbD Serotec) was also carried out on 1×10^6^ monocytes.

### Roche xCELLigence Chemotaxis Assay

**Figure 1:**
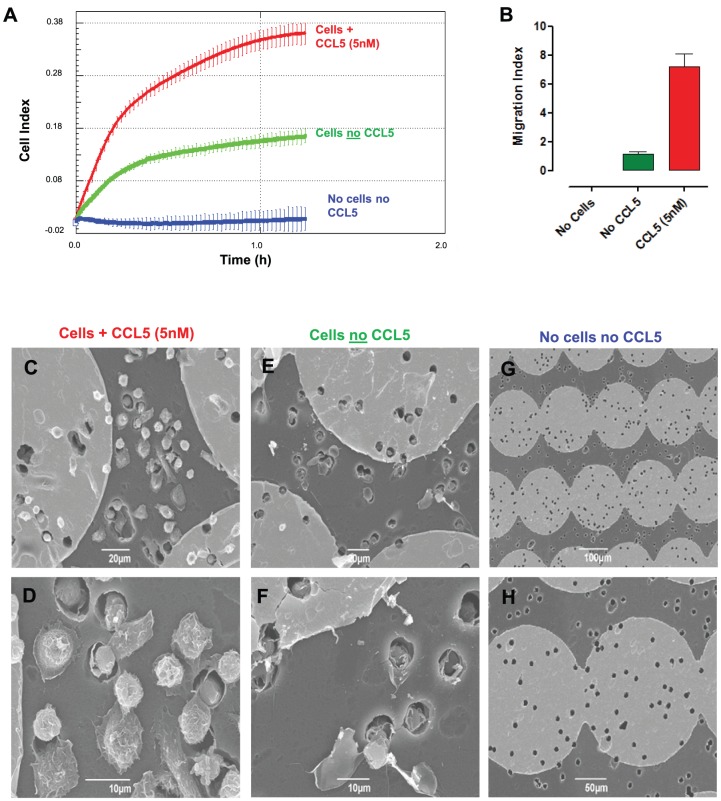
Real-time chemotaxis of Bio-Gel elicited macrophages to murine CCL5. (**A**) (**A**) Representative trace from the RTCA-DP software of 4×10^5^ Bio-Gel elicited macrophages from C57BL/6J male mice in the top chamber with murine CCL5 (5 nM) (red) or no chemokine (green) in the bottom chamber. A control of no macrophages and no chemokine was also run (blue). By 70 mins max CI had been reached, the plate was removed and membranes were cut and fixed with 2.5% gluteraldehyde and processed for scanning electron microscopy (SEM). Representative images are shown of macrophages adhered to the underside of filters in response to 5 nM murine CCL5 (**C & D**) no chemokine (**E & F**) or control where no cells or chemokine were added (**G & H**). Pores in the membrane are clearly visible in panel **H**. The number of cells adhered to individual gold discs on the underside of the membrane (seen as light grey regions in the SEM images) were quantitated (n = 1 experiment with technical replicates of each condition analysed) (**B**).

Chemotaxis assays were carried out with CIM-16 well plates and an xCELLigence RTCA-DP instrument (Roche Diagnostics, Burgess Hill, West Sussex, UK). Agonists were made to desired concentrations (final volume of 160 µl) and loaded in the lower wells of the CIM-16 plate. Following upper chamber attachment, the upper wells were filled with 50 µl pre-warmed chemotaxis buffer and the plate left for 30 mins at RT to pre-equilibrate. Bio-Gel, thioglycollate elicited and resident macrophages were resuspended to 8×10^6^ cells/ml in chemotaxis buffer and 50 µl cell suspension (4×10^5^ cells) was placed into the top wells. In contrast BMDM were resuspended at 1×10^6^ cells/ml in chemotaxis buffer. The addition of 4×10^5^ or 0.5×10^5^ cells per well was selected based on three different cell densities tested in response to increasing concentrations of CCL5 or CCL3 (data not shown). The assembled plate was transferred to the RTCA-DP machine and data was collected every 5 s over the course of 3000 sweeps (5 hours in total). As macrophages pass through the 8 µm pores towards a selected chemoattractant they adhere to the underside of the filter on which is embedded a gold micro-electrode. This produces a signal of electrical impedance which is reflected in the cell index as shown in the representative trace in (Figure 1A). Cell index is an arbitrary unit based upon the measured cell-electrode impedance derived by the software using the following calculation as described in reference [21]. Rising cell impedance therefore correlates to increasing numbers of migrated macrophages adhering to the underside of the filter which was confirmed by scanning electron microscopy (Figure 1C-H). Data were normalised to wells which received media alone and analysis for slope, max-min and area under the curve (AUC) were carried out with the RTCA Software 1.2.1. We found slope analysis to be a very useful measure for following the initial acceleration and migration of cells towards a wide range of chemoattractants. We believe that increasing slope of the curve to the point where maximum cell index is reached reflects true chemotaxis based upon profiles obtained for different leukocyte populations and analysis carried out with scanning electron microscopy at different time-periods. The AUC examines the complete time-course and therefore provides a combined measure for both migration and adhesion.

### Flow cytometry

Macrophages (1×10^6^) were washed once with PBS and re-suspended in FACS buffer (PBS; 2% FCS, 25 mM HEPES, 5 mM EDTA) containing CD16/CD32 FCγIIR blocking antibody (eBioscience, Wembley UK). Specific staining with the following antibodies was performed with appropriate isotype controls; F4/80 (AbD Serotec, Kidlington, Oxfordshire, UK), 7/4 (AbD Serotec), CCR2 (R&D systems), CCR5 (eBioscience) and MHC II (I-Ab; BD Biosciences) and analysed by flow cytometry to assess monocyte/macrophage numbers (Figure S1A & B). After a 30 min incubation at 4°C cells were analysed with Dako Cyan ADP flow cytometer (Beckman Coulter Ltd, High Wycombe, UK) and FlowJo software (Tree Star incorporation, Ashland, USA).

### Scanning Electron Micrscopy

For scanning electron microscopy, excised CIM-plate filters were fixed for ∼24 h with 2.5% glutaraldehyde in PBS. Fixed samples were rinsed several times with dH_2_O, dehydrated through a series of ethanols and critically point-dried. Filters were mounted bottom-side up (gold electrode surface) and sputter-coated with gold as previously described [22]. The samples were examined in a JOEL JSM 6390 scanning electron microscope. Quantification of macrophages was carried out in a blinded manner. Three gold discs per electrode were counted for all treatment groups.

### Primer design

*Ccr1, Ccr5, Ccr2* and *Actg1* (Actin G) primers were designed with Primer 3 (v.0.4.0) online software (http://frodo.wi.mit.edu/primer3/). They were designed to be 18–22 bp in size, have a T_m_ of 52–58°C, 40–60% GC content, amplicon product size of 90–150 bases, Max 3′ stability of 13 kcal/mol, three MaxPoly-Xs and Max Self and Max 3′ Self Complementarity as low as possible (Table 1). All primers were tested for hairpins, dimer and cross-dimer formation, palindromes and repeats with Premier Biosoft NetPrimer software (http://www.premierbiosoft.com/netprimer/index.html). Subsequently, all primers were BLASTed with the *Mus musculus* genome database to determine specificity.

**Table 1:** Primer sequences used in RT-PCR.

Gene	Sequence (5′->3′)	Length Bases	Accession Number	Primer exon location
*Ccr1 Fw*	CGAACTGTTACTTTTGGCATCATC	24	NM_009912.4	2
*Ccr1 Rv*	GTATAAGGCAGGCATGGAAGCT	22	NM_009912.4	2
*Ccr2 Fw*	CACCTTTGAGTGAGTGGGAAC	21	NM_009915.2	3
*Ccr2 Rv*	AGTGGACAGGGAAGAACATTT	21	NM_009915.2	3
*Ccr5 Fw*	AAGCTGAAGAGCGTGACTGATATCTA	26	NM_009917.5	2
*Ccr5 Rv*	GAATGGTAGTGTGAGCAGGAAGAG	24	NM_009917.5	2
*Actg1 Fw*	CCAACAGCAGACTTCCAGGATT	22	NM_009609.2	6
*Actg1 Rv*	CTGGCAAGAAGGAGTGGTAACTG	23	NM_009609.2	6

### RNA extraction and reverse transcription

RNA was extracted using the RNeasy® Mini kit (Qiagen) according to the manufacturer's instructions. The quality and concentration of RNA was obtained using a Nandrop ND-1000 Spectrophotometer (Nano Drop Technologies, *DE, USA*). RNA was reverse transcribed to DNA with the Qiagen® QuantiTect Reverse Transcription kit (Qiagen) according to the manufacturer's instructions.

### qPCR

cDNA samples were run on a qPCR platform to determine mRNA gene expression. The detection used for the experiments was Sybr® Green Jumpstart™ Taq ReadyMix™ (Sigma-Aldrich). Master-mixes were prepared for both the genes of interest (GOI) and the reference genes (Table 1 & 2). The qPCR thermal profile included one phase at 95°C for 5 min, second phase of 40 cycles at 95°C for 20 s, 60°C for 20 s and 72°C for 20 s, and last phase of 72°C for 10 min. Melt curve analysis was also performed on PCR products. Normalized fluorescence threshold was set to 1. Samples were run in duplicates in a RG-3000 thermal cycler (Corbett research, Manchester UK) and analysed with Rotor-gene 6.1 software.

**Table 2:** Chemokine receptor mRNA expression in Bio-Gel and thioglycollate elicited macrophages.

Chemokine receptor	Bio-Gel (ΔCt value)	Thioglycollate (ΔCt value)
*Ccr1*	4.07±0.35	5.7±0.09
*Ccr2*	3.39 ± 0.61	4.48.±0.09
*Ccr5*	4.55±0.24	7.75±1.12

### Statistical analysis

All data are reported as mean ± SEM of n observations. Statistical evaluation was performed using one-way or two way analysis of variance (ANOVA) (Prism5 GraphPad Software, San Diego, CA) followed by Dunnett's or Bonferroni multiple comparison posthoc test, taking a probability *P*<0.05 as statistically significant. Where two variables were analysed, a Student's *t*-test was applied.

## Results and Discussion

### Validation of a real-time macrophage chemotaxis assay across several murine macrophage populations

Macrophages are an ideal cell type for chemotaxis assays given that they migrate toward a wide range of chemoattractants and possess the ability to adhere to multiple surfaces [23], [24]. Figure 1A shows a typical trace of Bio-Gel macrophage chemotaxis and adhesion in response to murine CCL5. In the absence of both macrophages and CCL5 there was no signal, in the presence of macrophages but no CCL5 there was a low background level of increasing impedance (max CI of 0.17), however, when there was a concentration gradient of CCL5 from the bottom well to the top well, macrophage chemotaxis could clearly be detected (max CI of 0.37). In line with these observations, scanning electron microscopy of the filters from the same experiment confirmed a higher proportion of macrophages adhered to the underside of filters from wells with CCL5 (Figure 1C & D) in comparison to those with no chemokine (Figure 1E & F) or no chemokine with no cells (Figure 1G & H). This was also corroborated following quantification of macrophages adhered to the gold discs on the underside of the filter seen as the lighter grey regions in the scanning electron microscopy images (Figure 1B).

**Figure 2:**
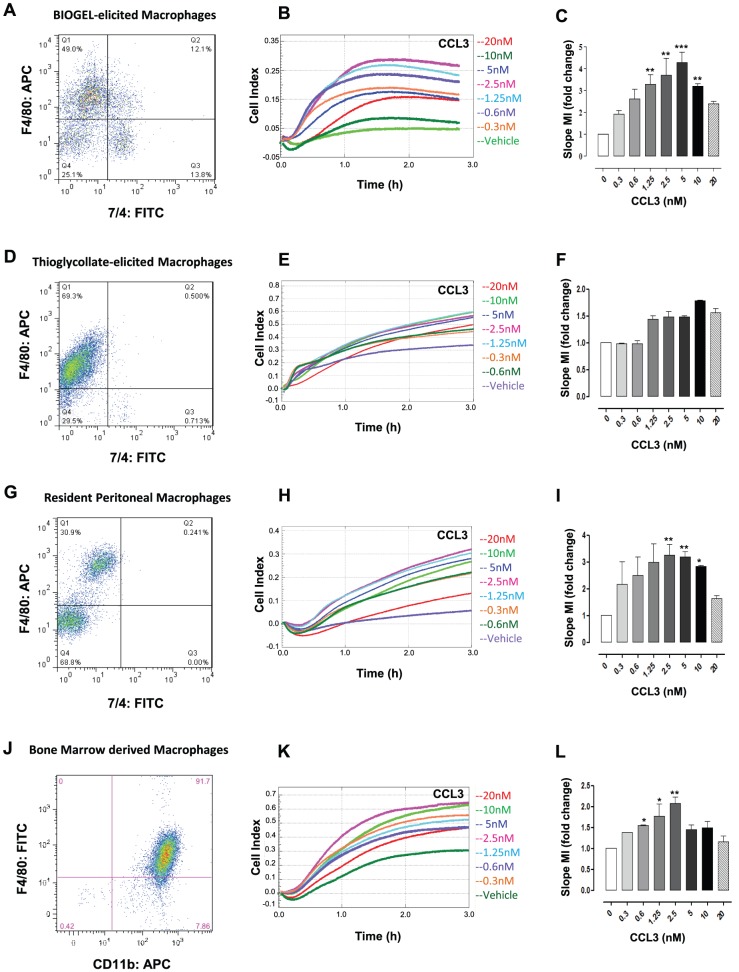
Real-time chemotaxis of different murine macrophage populations to murine CCL3. Panels **A, D, G & J** are representative FACS plots of the four different murine macrophage populations tested in the RTCA-DP system chemotaxis assay. (**A**) Injection with 2% Bio-Gel (polyacrylamide beads) typically yields 60–70% monocyte/macrophages which are collected after 4 days following the initial injection (assessed by staining with F4/80, 7/4 & CD11b antibodies). (**B**) 4×10^5^ Bio-Gel elicited peritoneal macrophages were allowed to migrate toward indicated concentrations of murine CCL3 for 3 h. (**C**) Migration was measured for each curve of each concentration using slope (set between 15–60 mins) analysis. (**D**) Injection with 4% Thioglycollate generates 70% F4/80^+^ 7/4^−^ macrophages which were harvested 4 days post injection. (**E**) 4×10^5^ Thioglycollate-elicited peritoneal macrophages were allowed to migrate toward indicated concentrations of murine CCL3 for 3 h (**G**). (**F**) Migration was measured for each curve of each concentration using slope analysis. (**G**) Lavage of the peritoneal cavity in non-elicited conditions yields ∼30% F4/80^hi^ resident peritoneal macrophages. (**H**) 4×10^5^ Resident peritoneal macrophages were allowed to migrate toward indicated concentrations of murine CCL3 for 3 h. (**I**) Migration was measured for each curve of each concentration using slope analysis. (**J**) Bone marrow cells cultured over 7 days in the presence of L929 conditioned media +10% FBS generated a 92% pure population of macrophages which expressed both CD11b and F4/80. (**K**) 0.5×10^5^ BMDM were allowed to migrate toward indicated concentrations of murine CCL3 for 3 h. (**L**) Migration was measured for each curve of each concentration using slope analysis. Data are shown as migration index (i.e. fold change over media alone) for slope analysis. Data are the mean ± SEM of 3–7 independent experiments, each concentration with 2–3 technical replicates per experiment. Statistical analysis was performed by one-way ANOVA and Dunnett's multiple comparison post-test. *, p<0.05, **, p<0.01, ***, p<0.01 relative to media alone.

Reviewing the literature relating to primary murine macrophage chemotaxis, BMDM and peritoneal macrophages are the most widely used cell populations which is unsurprising given that they make up the two most convenient sources of primary murine macrophages. In terms of yield, 2–3 million resident peritoneal macrophages can be recovered from the cavity. The use of Bio-Gel or Brewer's thioglycollate broth as eliciting agents can increase the yield to 10–15 million macrophages/cavity. A study carried out by Turchyn *et al*., examined phenotypic and functional differences between murine resident and induced peritoneal macrophages. The study reported specific markers including CD115 and CD62L to be highly expressed on peritoneal elicited macrophages compared to resident macrophages [25]. In contrast F4/80 was shown to be highly expressed on resident macrophages. In terms of inflammatory cytokine production, studies have shown that resident macrophages generate more IL-6 following *ex-vivo* stimulation with LPS compared to thioglycollate elicited macrophages [26]. In contrast the use of casein as an eliciting agent was shown to promote the production of IL-1β in macrophages following LPS stimulation, with no production observed in resident macrophage [25]. Other differences including antigen presentation and phagocytosis have also been reported [27], [28]. Collectively these functional differences highlight the need for a comparative analysis to be carried out on the migratory capacity amongst different macrophage populations. We therefore chose to validate this real-time chemotaxis assay with a range of primary murine macrophages including; Bio-Gel (Figure 2A) and thioglycollate (Figure 2D) elicited peritoneal macrophages, non-elicited resident peritoneal macrophages (Figure 2G) and BMDM (Figure 2J). Bio-Gel elicited macrophages which we identify as F4/80^+^ 7/4^low^ (Figure 2A) displayed a rapid dose dependent response to CCL3, with max cell index of 0.32 reached by 80 min (Figure 2B). Bio-Gel elicited macrophages in media alone recorded a background impedance of 0.05 (Figure 2B). Analysis by slope indicated a bell shaped dose response (Figure 2C) with maximal MI achieved at 5 nM, in line with published values generated with the modified Boyden chamber [8]. In contrast thioglycollate elicited macrophages (F4/80^+^ 7/4^−^ Figure 2D), displayed no dose dependent migration to CCL3 which was confirmed with slope analysis (n = 4 independent thioglycollate elicited macrophage preparations) (Figure 2E-F). Furthermore, thioglycollate elicited macrophages in media alone recorded a background cell impedance of 0.35 at 3 hours indicating a high level of non-directed migration (Figure 2E). This observation clearly highlights that although a potent eliciting agent, the use of thioglycollate does not provide an optimal source of peritoneal macrophages for this chemotaxis assay when compared to Bio-Gel elicited macrophages. Resident peritoneal macrophages which are F4/80^high^ (Figure 2G) displayed a dose dependent migration towards increasing concentrations of CCL3 (Figure 2H-I). In comparison to Bio-Gel elicited macrophages, both resident and thioglycollate macrophages generated a sustained cell index signal across time, consistent with a more adherent phenotype for these cells. BMDM (CD11b^+^/F4/80^+^) provide a homogenous population of macrophages compared to all three peritoneal phenotypes described, which can provide an effective means of reducing the number of animals required to carry out research of this type (Figure 2J). BMDM showed a rapid dose dependent migration toward CCL3 with max cell index of 0.64 reached by 140 min, however, a background impedance of 0.3 at 3 hours was also recorded with max MI at 2.5 nM (Figure 2K). Analysis by slope indicated a bell shaped dose response (Figure 2L). There are limitations to the use of BMDM, principally the time it takes to generate them and the requirement of activating with cytokines to induce certain receptors. The real-time chemotaxis assay shown in Figure 2 exemplifies the key differences in migration and adhesion profiles of the macrophage populations tested, which would be overlooked in conventional single time-point chemotaxis assays.

**Figure 3:**
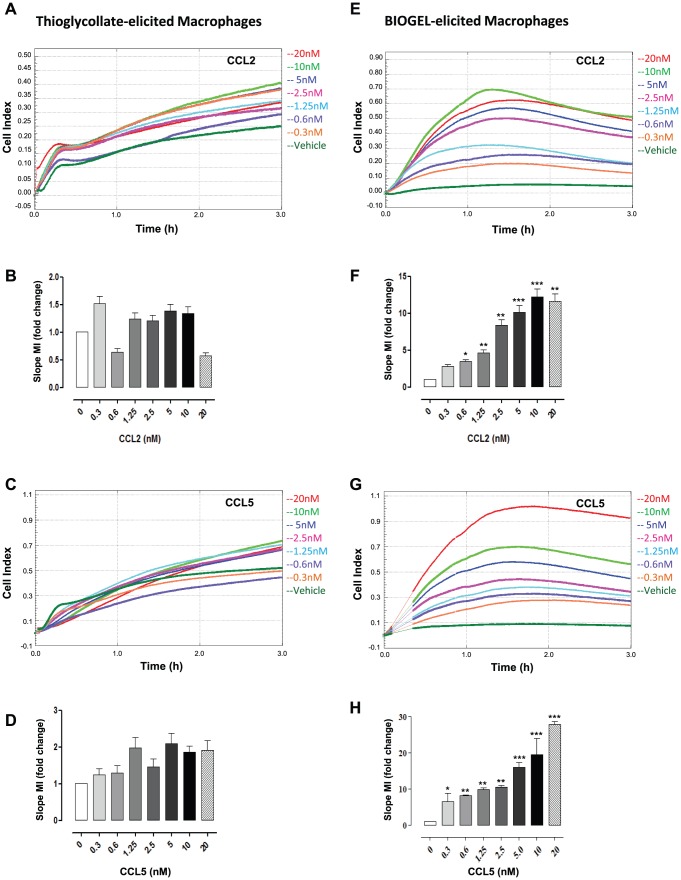
Bio-Gel and thiogclycollate elicited macrophage chemotaxis to CCL2 and CCL5. Thioglycollate and Bio-Gel elicited macrophages (4×10^5^ total) were allowed to migrate toward indicated concentrations of murine CCL2 (**A & E**) and CCL5 (**C & G**) for 4 h. Migration was measured for each curve of each concentration using slope analysis (**B** & **F- D** & **H**). Data are shown as migration index (i.e. fold change over media alone) for each parameter of analysis. Data are expressed as mean ± SEM of 3–5 independent experiments, each concentration with two technical replicates per experiment. Statistical analysis performed by one-way ANOVA and Dunnett's multiple comparison post-test. *, p<0.05, **, p<0.01, ***, p<0.01 relative to media alone.

The marked difference in behaviour of thioglycollate elicited macrophages compared to Bio-Gel elicited macrophages warranted further investigation. We therefore evaluated responses of both elicited macrophage populations to other CC chemokines. Analysis by slope indicated CCL2 and CCL5 caused significantly enhanced migration and adhesion in Bio-Gel elicited macrophages with increasing concentrations of chemokine (Figure 3E-H). In marked contrast, thioglycolate elicited macrophages displayed no dose-dependent response to CCL2 or CCL5 and recorded a high level of background impedance in wells containing media alone (Figure 3A-D).

To appreciate whether the migratory differences observed between Bio-Gel and thioglycollate elicited macrophages could in part be due to differences in chemokine receptor expression or activation we examined several markers at both mRNA and protein level. We found no differences in the receptor expression of CCR1, −2 and −5 both at mRNA (Table 2) and protein level (Figure S1D-E/J-K). The activation marker MHC II which has been widely reported to be up-regulated on recruited macrophages [23] was expressed at similar levels on both Bio-Gel and thioglycollate elicited macrophages (Figure S1C & I). Representative images taken of Bio-Gel and thioglycollate elicited macrophages show that thioglycollate macrophages are larger in size and have a greater number of protrusions (Figure S1F & L). Clearly, further studies will be required to fully elucidate differential chemokine receptor signalling in different elicited murine macrophage populations.

### Real-time macrophage chemotaxis in response to CC chemokines is Gαi signaling dependent and requires cytoskeletal re-arrangement

**Figure 4:**
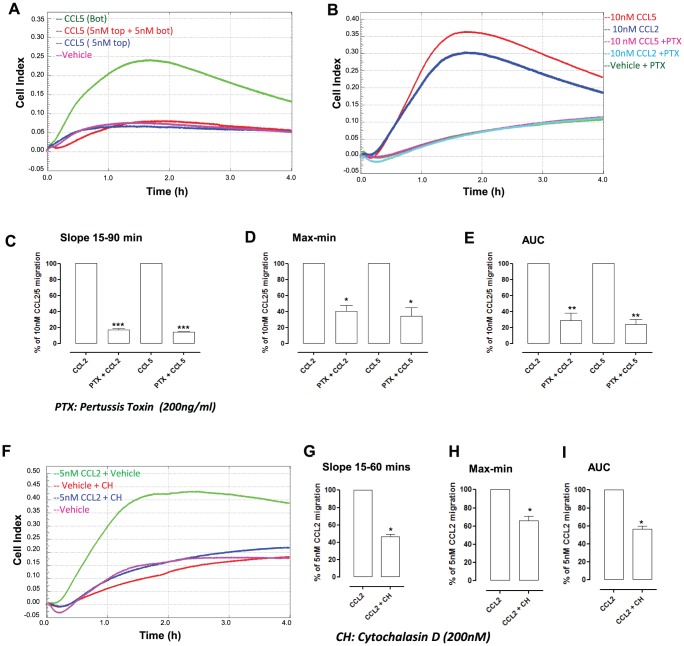
Demonstration of chemotaxis and its inhibition by Pertussis toxin (PTX) and cytochalasin D. To test that the assay was detecting true macrophage chemotaxis, rather than chemokinesis or fugetaxis, the following three conditions were tested; CCL5 added to the bottom well alone (*test chemotaxis*), equal concentrations (5 nM) of murine CCL5 added to both top and bottom wells (*test chemokinesis*) or CCL5 added to the top well alone (*test fugetaxis*) (**A**) Single trace of a single experiment with four technical replicates shows Bio-Gel elicited macrophages migrate towards murine CCL5 (green), but display no chemokinesis (red) or fugetaxis (blue). Control (pink) had chemotaxis buffer alone in the bottom well. Bio-Gel elicited macrophages were pre-incubated for 90 mins at 37°C with PTX (200 ng/ml). (**B**) A representative trace shows that pre-treatment of Bio-gel elicited macrophages with PTX significantly inhibits chemotaxis toward both murine CCL2 and CCL5. Data are shown as a percentage of 10 nM CCL2 or CCL5 induced migration for (**C**) Slope (**D**) Max-min CI and (**E**) AUC. Data are the mean ± SEM of three independent experiments with 3–4 technical replicates per experiment. Vehicle alone was omitted in these sets of experiments in order to have all five conditions on a single CIM-16 plate. (**F**) Bio-Gel elicited macrophages were pre-incubated for 90 mins with either 200 nM cytochalasin D, an inhibitor of F-actin polymerisation. A representative trace is shown (**F**). Data are shown as a percentage of 5 nM CCL2 induced migration for (**G**) Slope (**H**) Max-min CI and (**I**) AUC. Data are the mean ± SEM of four independent experiments with 3–4 technical replicates per experiment. Statistical analysis was performed by one-way ANOVA and Dunnett's multiple comparison post-test. *, p<0.05, **, p<0.01, ***, p<0.01 relative to CCL2 or CCL5 alone.

We next wanted to confirm that the system was truly detecting macrophage chemotaxis as opposed to chemokinesis or fugetaxis. To address this we set up the following three conditions; CCL5 added to the bottom well alone (to test chemotaxis), CCL5 added to the top well alone (to test fugetaxis) or equivalent concentrations of CCL5 in both top and bottom wells (to test chemokinesis). Figure 4A reports that only Bio-Gel macrophage chemotaxis toward murine CCL5 was detected with no evidence of either chemokinesis or fugetaxis. Chemokine receptors are G-protein coupled seven-transmembrane receptors which induce chemokine signalling via Gαi. It is well documented that pertussis toxin (PTX) can inhibit chemokine signalling [29] and thus chemotaxis in modified Boyden chamber assays. We therefore tested the inhibitory actions of PTX on Bio-Gel elicited macrophages in response to murine CCL2 and CCL5. Pre-treatment of Bio-Gel elicited macrophages with PTX (200 ng/ml) abrogated macrophage chemotaxis and adhesion toward CCL2 and CCL5 (a representative trace is shown in Figure 4B). Pooled data from three independent experiments revealed highly significant inhibition (p<0.001) as measured by slope, max-min and AUC analysis (Figure 4C-E). PTX (200 ng/ml) pre-treatment of resident peritoneal macrophages was also shown to inhibit chemotactic responses to 10 nM CCL2 as measured by slope, max-min and AUC analysis (Figure S2A-D).

To further validate this novel system we tested the need for actin cytoskeletal re-arrangement for directed migration of leukocytes toward chemoattractants [1], [30], [31]. Pre-treatment of macrophages with cytochalasin D which inhibits F-actin polymerisation, significantly inhibited macrophage migration and adhesion toward 5 nM murine CCL2 as shown in a representative trace (Figure 4F) and by slope, max-min and AUC analysis of three independent experiments (Figure 4G-I). A partial but significant inhibition was also observed with the ROCK (Rho-associated, coiled-coil containing protein kinase 1) inhibitor, Y-27632 (data not shown).

### Analysis of macrophage chemotaxis towards a range of chemoattractants

**Figure 5:**
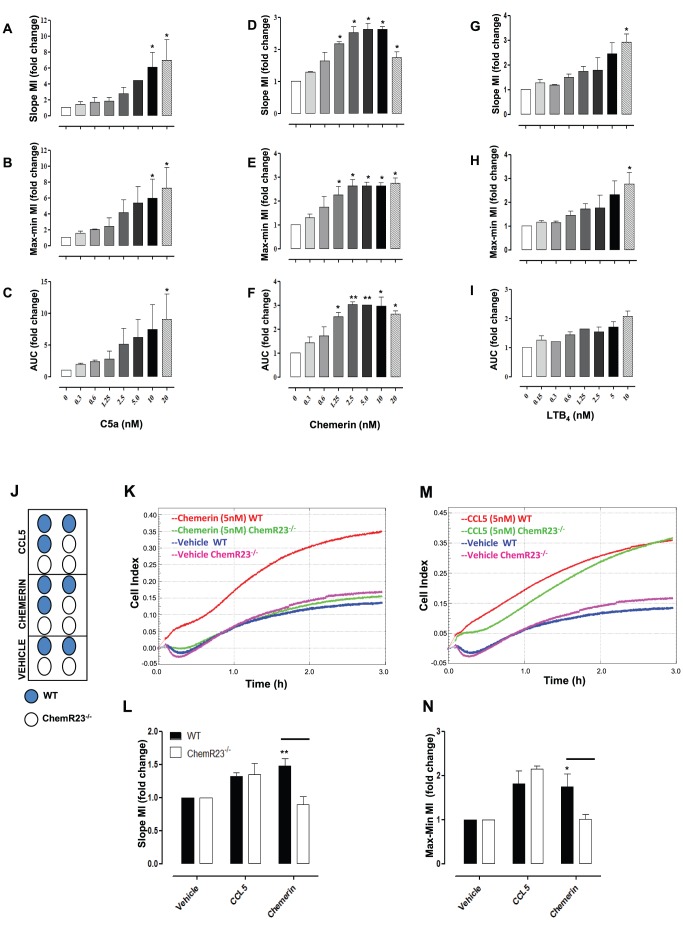
Bio-Gel elicited macrophage chemotaxis to non CC chemokine chemoattractants. Bio-Gel elicited macrophages from C57BL/6J (4×10^5^ total) were allowed to migrate toward indicated concentrations of murine C5a, chemerin and LTB4 for 4 h. Migration was measured for each curve of each concentration using slope (**A, D, G**), max-min (**B, E, H**) and AUC (**C, F, I**) analysis. Data are shown as migration index (i.e. fold change over media alone) for each parameter of analysis. Data are expressed as mean ± SEM of 3–5 independent experiments, each concentration with two technical replicates per experiment. Statistical analysis performed by one-way ANOVA and Dunnett's multiple comparison post-test. *, p<0.05, **, p<0.01, ***, p<0.01 relative to media alone. (**J**) A schematic representation of the plate designed used to test whether the chemotactic effects of chemerin are ChemR23 dependent. Representative traces of Bio-Gel elicited macrophages from ChemR23^-/-^ and WT controls in response to (**K**) chemerin and (**L**) CCL5 are shown. Migration was measured for each curve using slope (**M**) and Max-min CI (**N**). Data are the mean ± SEM of three independent experiments with 2–4 technical replicates per experiment. Statistical analysis was performed by two-way ANOVA and Bonferroni multiple comparison post-test. *, p<0.05, **, p<0.01, relative to WT.

Murine Bio-Gel elicited macrophages were allowed to migrate towards increasing concentrations of other murine chemoattractants including complement C5a, leukotriene B4 (LTB4) and chemerin, a potent chemoattractant of antigen presenting cells [32] (Figure 5A-L). Chemerin displayed similar to effects to CCL3, inducing a bell- shaped dose response curve when analysed using all three parameters (Figure 5D-F). Analysis by slope, max-min and AUC also exemplified robust chemotactic effects for C5a and LTB4 in this system (Figure 5A-C/G-I). To further exemplify the use of this real-time chemotaxis assay as a functional readout we tested macrophages from receptor knockout mice. Chemerin has been previously reported to bind ChemR23 and promote macrophage chemotaxis as assayed using a modified Boyden chamber [32], [33]. We used Bio-Gel elicited macrophages from ChemR23 KO mice and Sv129 controls to confirm that the chemotactic effects of chemerin are dependent on ChemR23. The 16 well plate design shown in Figure 5J was used with 5 nM CCL5 as a positive control. Representative traces from a single experiment show that the chemotactic effects of chemerin were ablated in ChemR23 KO macrophages (Figure 5K) with no effect on chemotactic responses to CCL5 (Figure 5M). The lack of chemotaxis to chemerin was also shown to be statistically significant following analysis with slope and max-min in three independent experiments (Figure 5L-N).

### Human monocyte chemotaxis reveals distinct migration behaviour amongst donors

**Figure 6:**
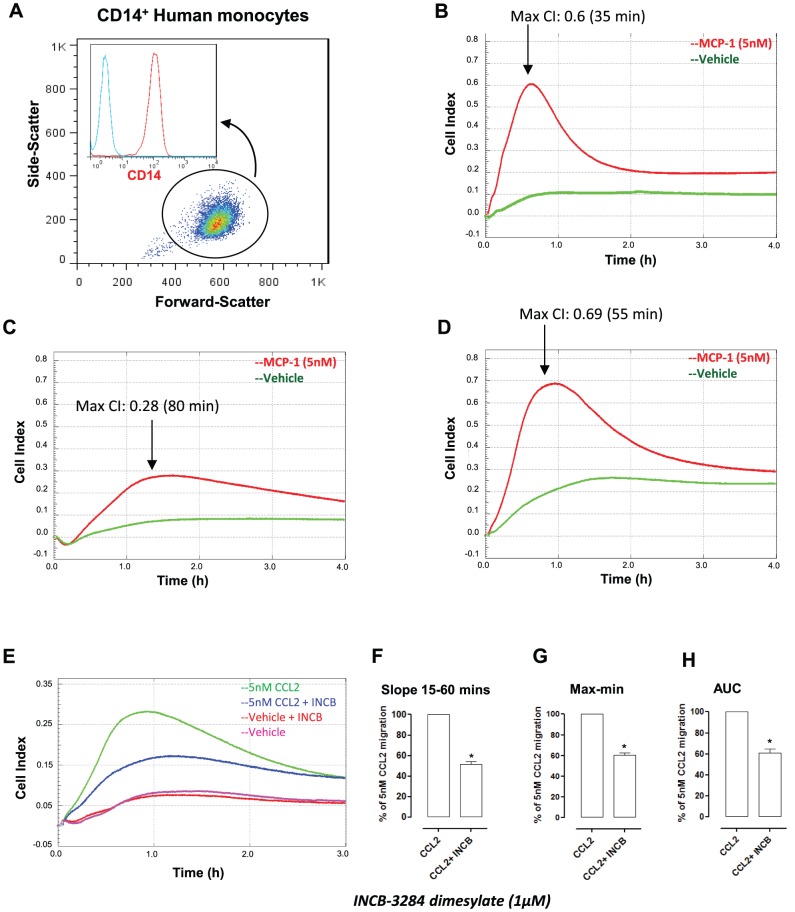
Analysis of chemotaxis responses in human monocytes reveals distinct donor to donor variation. Panel (**A**) shows a representative FACS plot of human monocytes following isolation by CD14 positive selection (Inset panel red = CD14^+^; blue =  isotype control Ab). Panels **B, C,** and **D** are individual traces from three independent donors measuring monocyte (4×10^5^ total) chemotaxis toward 5 nM human CCL2. (**E**) The inhibitory action of an anti-CCR2 drug (INCB-3284 dimesylate) was also tested with human monocytes. Human monocytes were pre-incubated with INCB-3284 (1 µM) for 90 mins at 37°C and allowed to migrate toward 5 nM CCL2 for 3 h. Data are shown as a percentage of 5 nM human CCL2 induced migration for (**F**) Slope (**G**) Max-min CI and (**H**) (AUC). Data are the mean ± SEM of three independent experiments with four technical replicates per experiment. Statistical analysis was performed by one-way ANOVA and Dunnett's multiple comparison post-test. *,p<0.05 relative to CCL2 alone.

We next wanted to examine whether chemotaxis of other primary myeloid cells could be detected using this system. Human CD14^+^ monocytes (Figure 6A) isolated from whole blood by positive selection were tested in response to 5 nM human CCL2. Figure 6B-D show individual traces from three independent human monocyte donors. The kinetics of chemotaxis and adhesion from all three monocyte donors shows strong variation in terms of the maximum cell index and the time taken to reach it, emphasising the biological variation amongst human donors. Collectively these results highlight the advantage of this real-time chemotaxis assay given that this data would be missed in conventional end-point assays. The drug INCB-3824 dimesylate was also tested to see whether CCL2 induced migration of CD14^+^ human monocytes could be blocked by a CCR2 receptor antagonist. Pre-treatment of monocytes with INCB-3824 inhibited CCL2-induced macrophage migration and adhesion (Figure 6E). Analysis by slope, max-min and AUC all showed that this inhibitory action was statistically significant (Figure 6F-H).

To date a limited number of studies have reported the use of transformed cell lines in the xCELLigence CIM-Plate 16 device to monitor cell migration [34]–[36]. In the study performed by van Giles and colleagues the authors reported the use of this technology to monitor the migration of RAW cells to CCL2. The authors demonstrated after 16 hours RAW cells reached a max CI of 0.7 [29]. In contrast we have shown in primary Bio-Gel elicited macrophages the time taken to reach max CI occurs far more rapidly (1.5 hours) and to a similar magnitude, highlighting key differences in the responsiveness of primary and transformed cells detected in this system.

To our knowledge this is the first study to comprehensively validate and optimise the xCELLigence real-time migration assay for primary murine and human macrophages. We believe that the xCELLigence real-time migration assay offers a rapid and reproducible system to monitor macrophage chemotaxis. Continuous collection of impedance measurements over a user defined time period also offers valuable kinetic information relating to both cell migration and adhesion. Quantification is also far more rapid and accurate compared to the modified Boyden chamber where multiple images are required followed by staining and counting which can also introduce operator bias. A common technical issue also associated with the modified Boyden chamber is the loss of wells resulting from pipetting error, during the attachment of the upper and lower chambers or when transferring the plate to the incubator. In contrast we have experienced minimal loss of wells with the real-time chemotaxis assay. The RTCA-DP software allows operator inspection of individual well traces and hence identifies wells with poor electrical conduction or artefacts most likely caused by bubbles. The system also provides a platform to monitor cells from different genetic backgrounds and disease models for comparison as shown in the experiments of Figure 5J-N. There are some limitations; the fixed pore size of 8 µm does make it challenging to monitor different leukocyte populations given that we generally use 5 µm pores for modified Boyden chamber analysis of human CD14^+^ monocytes and neutrophils and 3 µm for T cells. The system in its current format is also limited to studying adherent cells, as we found no changes in CI were detected following T cell chemotaxis in response to a range of CXCL12 concentrations (data not shown). The 16 well format of the CIM-16 plate limits the number of technical replicates and conditions used. However the system does have the capacity to run three CIM-16 plates independently or simultaneously providing users with the potential of a 48-well design, allowing the derivation of more accurate EC_50_ or IC_50_ values for agonists and antagonists.

In summary, our paper reports a robust, reproducible and easily quantitated functional read out for primary monocyte/macrophage chemotaxis. We show that different murine macrophage populations exhibit marked differences in their migratory behaviour to CC chemokines that are not related to chemokine receptor expression. Now that we have validated this methodology we can address specific questions in macrophage biology and undertake mechanistic studies using real time chemotaxis as a readout.

## Supporting Information

Figure S1 **Flow cytometric analysis of chemokine receptor expression in Bio-Gel and thioglycollate elicited macrophages.** (**A**) Bio-Gel and Thioglycollate elicited macrophages were analysed by FACS for CCR2 and CCR5 expression levels. Bio-Gel and thioglycollate macrophages were initially gated by forward (FSC) and side (SSC) scatter (**A &G**) followed by F4/80 and CDllb expression (**B & H**). Macrophages positive for both CDllb and F4/80 were then assessed for MHC II (**C & I**), CCR2 (**D & J**) and CCR5 (**E & K**) expression (Isotype controls; blue) (Mean fluorescence intensity [MFI] values are provided). Data are representative of two independent macrophage isolations. Representative image of (**F**) Bio-Gel and (**J**) thioglycollate elicited macrophages stained with methyl blue and eosin are shown at 40× magnification.
(EPS)

Figure S2 **Pertussis toxin (PTX) inhibition of resident peritoneal macrophage chemotaxis.** Resident peritoneal macrophages were pre-incubated for 90 mins at 37°C with PTX (200 ng/ml). A single trace shows that pre-treatment of resident macrophages with PTX inhibits chemotaxis toward murine CCL2.
(EPS)
